# Applicability of Automated Cell Counter with a Chlorophyll Detector in Routine Management of Microalgae

**DOI:** 10.1038/s41598-018-23311-8

**Published:** 2018-03-21

**Authors:** Toshiyuki Takahashi

**Affiliations:** Department of Chemical Science and Engineering, National Institute of Technology, Miyakonojo College, Miyakonojo, Japan

## Abstract

Microalgae have attracted attention for several industrial applications, but all such applications demand culture quality because of their sensitivity to environmental changes. Although simplicity, speed, and accuracy are important to assess algal cultures, researchers have expended vast amounts of labor to monitor algal health using hemocytometry. Along with its user bias, quantifying the cell status aside from the cell density is not easy. This paper describes the easy and rapid evaluation of algal number and status using an image-based cell counter (Countess II FL; Thermo Fisher Scientific Inc.) with a fluorescent filter for chlorophyll. Unlike mammalian cultured cells larger than microalgae, it is not easy for a low-resolution camera alone to distinguish microalgae from grimy spots and microbubbles on counting plates. To assess this method’s performance, freshwater/marine microalgae and environmental samples were evaluated using the instrument. Results reveal that an instrument with a fluorescence filter can distinguish microalgae from other particles more precisely than a device with no filter. Values obtained using the instrument were not significantly different from those obtained using hemocytometry. Moreover, the cell counter, but not hemocytometry, can qualify the algal status. Results demonstrate that this system, which has no user bias, can contribute to algal assessment.

## Introduction

**Figure 1:**
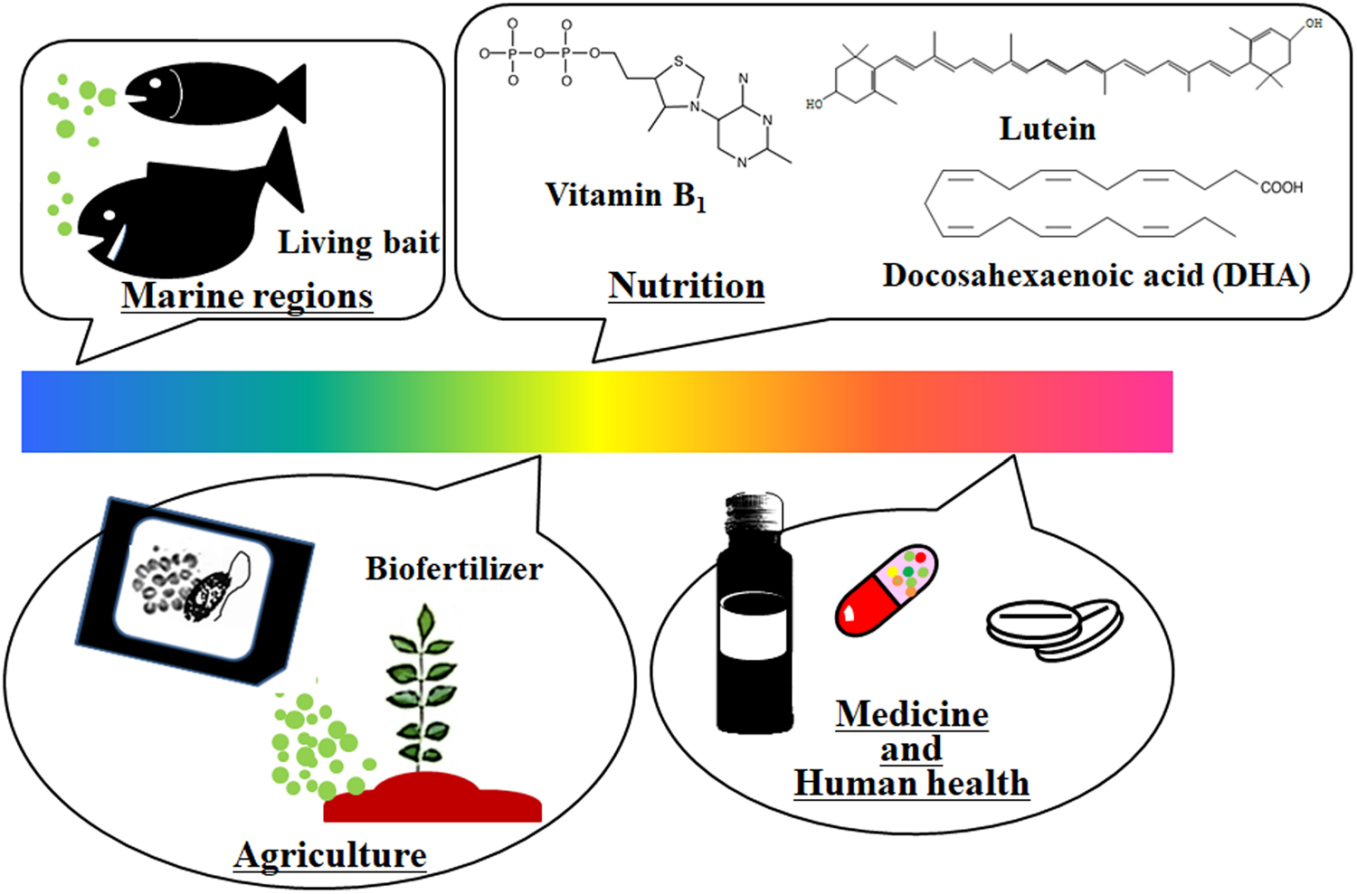
Potential of microalgae for industrial applications.

Broadly diverse biotechnologies have been likened recently to colors based on their use applications^1^. Red biotechnologies are associated with health and medical areas. Yellow ones are related to food and nutrition science. Blue ones are applicable to aquaculture, coastal, and marine areas. Green ones are agricultural and environmental applications^2–5^. White ones are related to industrial processes including chemical production and others. Although no explanation of the important roles of phytoplankton in aquatic ecosystems is necessary, several microalgae have been used for development in other areas. Microalgae might be assigned to most of the biotechnology categories presented above^2,3,6–8^ (Fig. 1). What is certain is that industrial application of algae demands the selection of useful algal species, the evaluation of algal features, and the assessment of their qualities in culture^9^. The guarantee of algal attributes is particularly important because microalgae are sensitive to environmental changes^10^. Consequently, routine control and management of algal quality in culture is the *sine qua non* for industrial applications.

Conventionally, microscopy, hemocytometry, and UV-Vis spectroscopy have been used to evaluate both algal cultures and environmental samples. Spectrofluorometry and flow cytometry (FCM) have also been used for detailed analyses^11–16^. Actually, microscopy and hemocytometry can elucidate characteristics of algae. They are useful to keep a tally of the number of algae manually. Vast amounts of time for obtaining data have been expended for studies based on these microscopic techniques. However, using these techniques renders studies vulnerable to user bias and misuse of hemocytometry^17^. Moreover, it might not be easy to quantify the cell status beyond the cell density. UV-Vis spectroscopy and spectrofluorometry might be useful to evaluate the overall cell status based on chlorophyll properties. Actually, FCM, analogously with spectrofluorometry, can detect the chlorophyll fluorescence of algae and can evaluate several properties of individual alga aside from chlorophyll. It is also useful to ascertain the number of algae. However, specialized skills to set up measurement parameters at the outset of FCM are necessary to perform a selective count and an accurate evaluation of each cell of interest. The salient difficulty might be the fact that these techniques cannot capture cells of interest visually.

Commercially available automated cell counters (TC20 Automated Cell Counter from Bio Rad Laboratories Inc., DigitalBio from NanoEnTek Inc., Cell Counter model R1 from Olympus Corp., and others) have been used for standard target cells such as cultured animal cells, e.g., human, rat, and mouse cells, which are generally larger than microalgae. These technologies help researchers shorten their routine tasks, including the checking of cell health and cell numbers. Attached cameras of almost all cell counters, however, are insufficient for microalgae detection because of their low-resolution images. Standard cameras of each cell counter used for visualization-based methods sometimes have difficulty discriminating small cells such as microalgae from other particles such as debris, small stains and spots, and microbubbles in a counting chamber. Although impedance-based methods using the Coulter principle^17^, light scatter-based methods such as FCM^9,12,13,16^ and visualization-based methods such as several cell counters^18^ are used for detection of cells of several types, accurate detection of small cells such as microalgae remains as a challenge for study in this field.

Envisioning the eventual use of algae for industrial applications, it is important to evaluate the algal status as routine work. Considering cost-performance, this study particularly examined the detection of microalgae using chlorophyll autofluorescence. No specific reagent is necessary for chlorophyll detection compared to other parameters such as DNA contents and lipids. This paper presents a method of easy and rapid evaluation of the number of algae and algal status using a compact, automated, and image-based cell counter (Countess II FL cell counter; Thermo Fisher Scientific Inc.) with a fluorescence filter for chlorophyll fluorescence. To assess the performance and precision of this method, several cultured microalgae were evaluated using a cell counter. Results show that the cell counter with the fluorescence filter can distinguish algae from other particles more precisely than that with no fluorescence filter, even for 2-μm-diameter microalgae. In addition to precise quantification of microalgae, the device can simultaneously evaluate the algal status based on chlorophyll fluorescence. This method relieves researchers from laborious and time-consuming routine tasks including the checking of algal health and numbers. This study also examined the performance potential of the cell counter on a range of detectable algae and photosynthetic plankton. It can contribute to the development of algal applications.

## Results

### Applicability of the imaging-based cell counter to microalgae detection and counting

**Figure 2:**
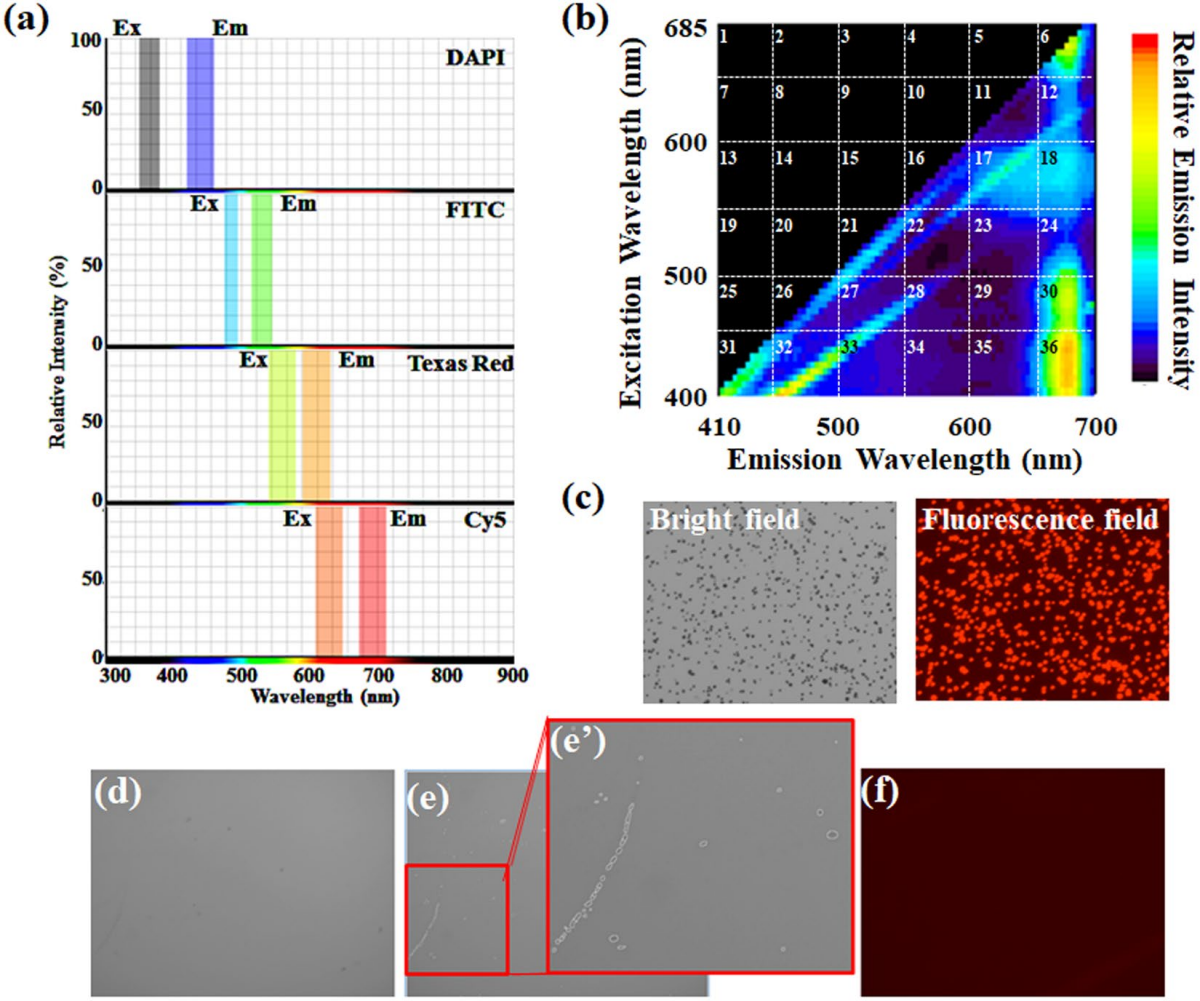
Fluorescence properties of the cell counter and microalgae. (**a**) An appropriate filter for the cell counter used for this study was examined using online software (SpectraViewer; Thermo Fisher Scientific Inc.). (**b**) Both excitation and emission spectra of *Chlorella*-like algae were obtained using spectrofluorometry. (**c**) Microalgal images were taken in the bright field image (left) and the corresponding fluorescence image (right) using the digital camera of the cell counter. Images (**d**–**f**: blank sample using sterilized water) from the cell counter are shown. Panels (d)–(e) and the magnified image (e’) of panel (e) are bright field images. Detected objects are highlighted with white circles. Panel (f) is the corresponding fluorescence image of panel (d). Detected objects are highlighted in panels (e) and (e’). By default, no original image from the cell counter had a scale bar.

This study evaluated the applicability of a cell counter (Countess II FL; Thermo Fisher Scientific Inc.) to detect microalgae such as *Parachlorella kessleri* (C-531 strain), *Chlorella*-like symbiotic algae (SA-1 strain) isolated from *Paramecium bursaria* and sea algae species. Brief species identification using PCR amplification and phylogenic clustering (Supplemental Fig. 1) revealed that the sea alga used for this study was a species related closely to a stramenopile alga, particularly algae in the class *Chrysophyceae* of the phylum *Stramenopiles*. The cell counter used for this study can use several fluorescence filters (Fig. 2a), which are useful for sorting out cells of interest from heterogeneous populations of cells. Figure 2a shows several fluorescence filters for representative fluorophores DAPI, FITC, Texas Red, and Cy5. Several metabolisms can be examined and evaluated using commercially available fluorophores: DNA-binding fluorophores such as DAPI are useful for cell cycle analysis; a fluorescent dye, BODIPY (4, 4-difluoro-1,3,5,7,8-pentamethly 4-bora-3a,4a-diaza-s-indacene), is useful for determination of neutral lipids, oil and other nonpolar lipids. Considering the cost-performance and availability for use in routine work, this study specifically emphasized autogenous chlorophyll fluorescence. Figure 2b presents a graph obtained using three-dimensional (3D) fluorescence excitation–emission matrix spectroscopy of *Chlorella*-like symbiotic algae from *P. bursaria*. The graph is presented in a grid pattern for descriptive purposes. Fluorescence emissions in 6, 12, 18, 24, 30, and 36 of the grid numbers included algal emissions at 680 nm, which has been used for chlorophyll fluorescence^9,19^. It is noteworthy that a greater or lesser degree of emission intensity reflects a difference in the excitation efficiency of chlorophyll molecules irradiated by light of each excitation wavelength. The result (Fig. 2b) shows that the best filter to detect chlorophyll is one that can detect fluorescence in No. 36 of the grid. However, a commercially available filter that can excite target cells at short wavelengths of 400–450 nm and which can simultaneously detect emissions at long wavelengths is rarely available. Therefore, this study used the fluorescence filter for Cy5 to detect microalgae. In principle, the filter can detect red fluorescence as depicted in No. 12 of the grid (Fig. 2b).

Analogously with FCM and other commercially available automated cell counters, the instrument used for this study can preset threshold levels of detection on size and/or the corresponding fluorescence intensity. This process helps the instrument to avoid falsely recognizing non-target particles as a cell of interest. To detect microalgae using the cell counter, all objects larger than 1 μm objects size were detected in this study. Microalgae of less than 5 μm diameter are generally too small for an attached camera of the cell counter. Although data integrity must be tested at this stage, this apparatus detected algae (Fig. 2c). A blank sample using sterile distilled water was measured just to be sure (Fig. 2d–f). Although no object was observed at a glance (Fig. 2d), insensible grime on the counting chamber was detected in bright field in response to a preset threshold level of detection sensitivity (Fig. 2e and e’). By contrast to the bright field image, no object was detected clearly in the fluorescence image (Fig. 2f).

**Figure 3:**
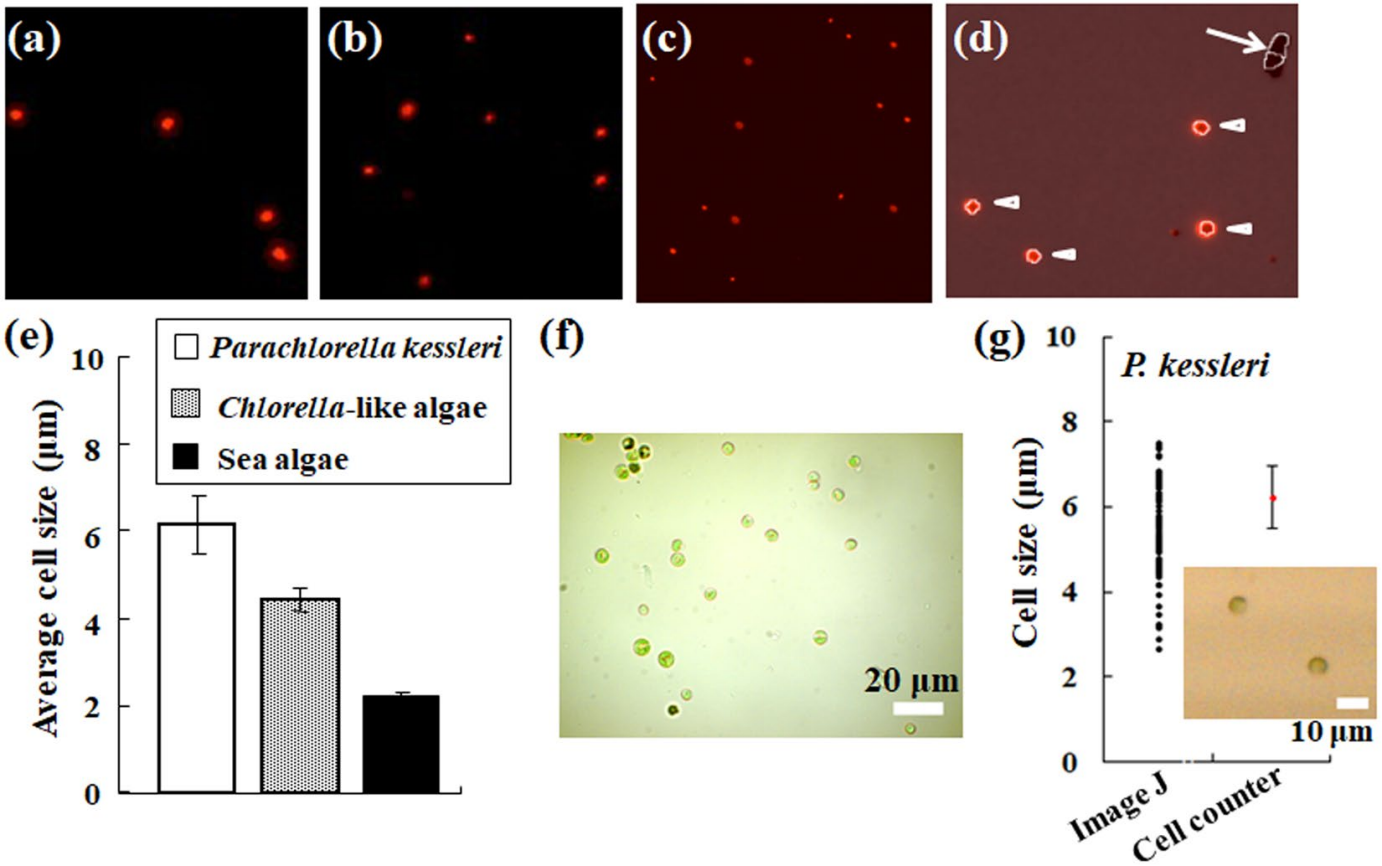
Detection and size measurements of microalgae using the cell counter with a fluorescence filter for Cy5. Chlorophyll fluorescence of *P. kessleri* (**a**), *Chlorella*-like symbiotic alga isolated from *P. bursaria* (**b**) and sea algae (**c**) were detected using the cell counter. (**d**) Example of algal detection using the specified algorithm of the cell counter. Here, *P. kessleri* was used as algae. The image merges a bright field image into the corresponding red fluorescence image. An arrow indicates signals without red fluorescence, which are detected only in the bright field image. By contrast, arrowheads show signals with red fluorescence, which are detected in both bright field and the corresponding fluorescence images. By default, no original image (**a**–**d**) from the cell counter has a scale bar. To make the algae more visible, each image (**a**–**d**) is shown at arbitrary magnification. (**e**) Size comparison of algal cells using the cell counter. (**f**) Micrograph of *Chlorella*-like symbiotic alga isolated from *P. bursaria* using light microscopy. The scale bar shows 20 μm. (**g**) Comparison of *P. kessleri* sizes (average ± standard deviation, red circle) using the cell counter with values obtained using microscopy and ImageJ software manually (each black circle). The scale bar in the inset image shows 10 μm.

In addition to cell detection, this cell counter can evaluate the size of the cells of interest. The cell counter can detect microalgae used for this study using the fluorescence detector (Fig. 3a–c). In response to a preset threshold level of detection sensitivity, the target alga was detected automatically using the cell counter (Fig. 3d). Each cell size of algae selected fluorescently using the Cy5 filter was estimated (Fig. 3e). All algae were smaller than 10 μm in size. The sea algae were almost uniformly of 2 μm in size. To verify the cell counter accuracy for cell size assessment, algae were observed using light microscopy. *Chlorella*-like algae (Fig. 3f), for instance, appear to have reasonable size for results obtained using the cell counter. Here, the measurement accuracy of algal size obtained using the cell counter was compared with data obtained using manual image analysis software such as ImageJ (Fig. 3g). Diameters of *Parachlorella kessleri* algae were calculated from each algal area that had been identified using ImageJ. Holding *P. kessleri* algae (inset image, Fig. 3g) up as an example, the diameters of *P. kessleri* (black circles in Fig. 3g) were estimated as a true circle because the circularity of individual alga obtained using ImageJ was approximately 0.92 ± 0.03. The average size and standard deviations of *P. kessleri* algae obtained using the cell counter (red circle in Fig. 3g) were included in the variation of the diameters using manual image analysis.

**Figure 4:**
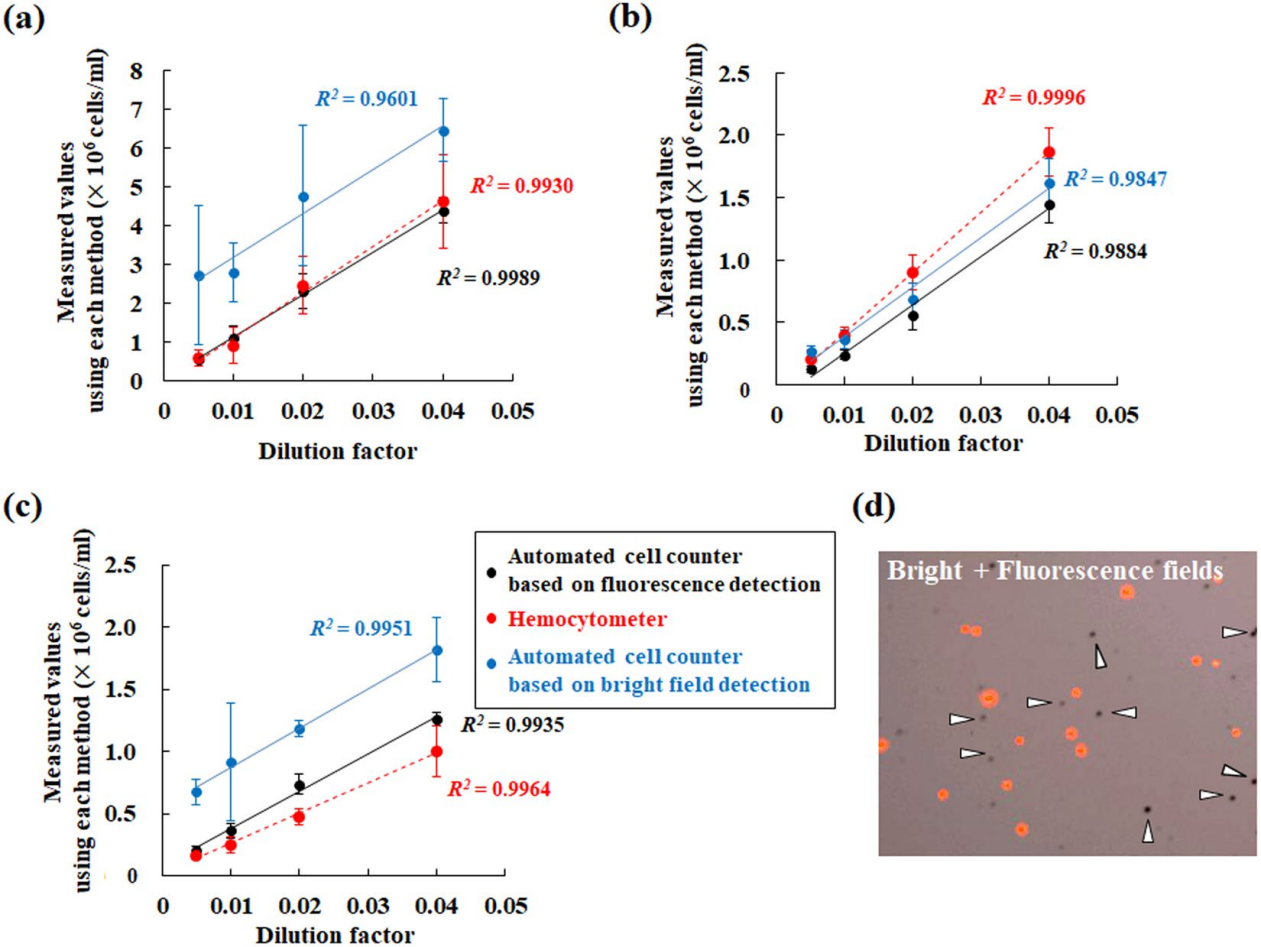
Comparison of measured values of microalgal densities obtained using the cell counter with those obtained using hemocytometry. Cell densities of *P. kessleri* (**a**), *Chlorella*-like symbiotic alga isolated from *P. bursaria* (**b**) and sea algae (**c**) were ascertained using the cell counter and a hemocytometer. Measured values obtained using the cell counter with (black solid line) or without the fluorescence filter (blue solid line), and those using the hemocytometer (red dotted line) are shown. (**d**) A merged image of a bright field image is shown with the corresponding fluorescence image. With no fluorescence filter, algal debris or grimy stains on the counting glass plate (arrowheads) might engender false recognition. By default, no original image from the cell counter has a scale bar.

To count the algae, this study used the cell counter described above, equipped with a fluorescence filter for Cy5, and did a dedicated reusable slide. Use of the reusable slide was based particularly on consideration of cost performance during routine application. Arbitrary concentrations of diluted algal samples were prepared. Then, the cell densities were ascertained using the cell counter and a hemocytometer. To compare cell count values of each microalga species obtained using the cell counter with those obtained using hemocytometry, the relation between each measurement value and each dilution factor is shown for each method (Fig. 4a–c). Here, values obtained respectively from the cell counter are expressed with and without the fluorescence filter for Cy 5. The values from the cell counter with the fluorescence filter (black solid line) were sufficiently similar to those obtained using hemocytometry (red dotted line). The coefficient of determination based on results obtained using the cell counter was higher than 0.9, even without the fluorescence filter. Values measured using the cell counter without the fluorescence filter (blue solid line), however, were occasionally different based on results obtained using hemocytometry (Fig. 4a and c). When not in use of any fluorescence filter, their error values might be attributable primarily to false recognition of non-cell debris, and to grimy stains and microbubbles on the counting glass plate (Figs 2d–f and 4d).

**Figure 5:**
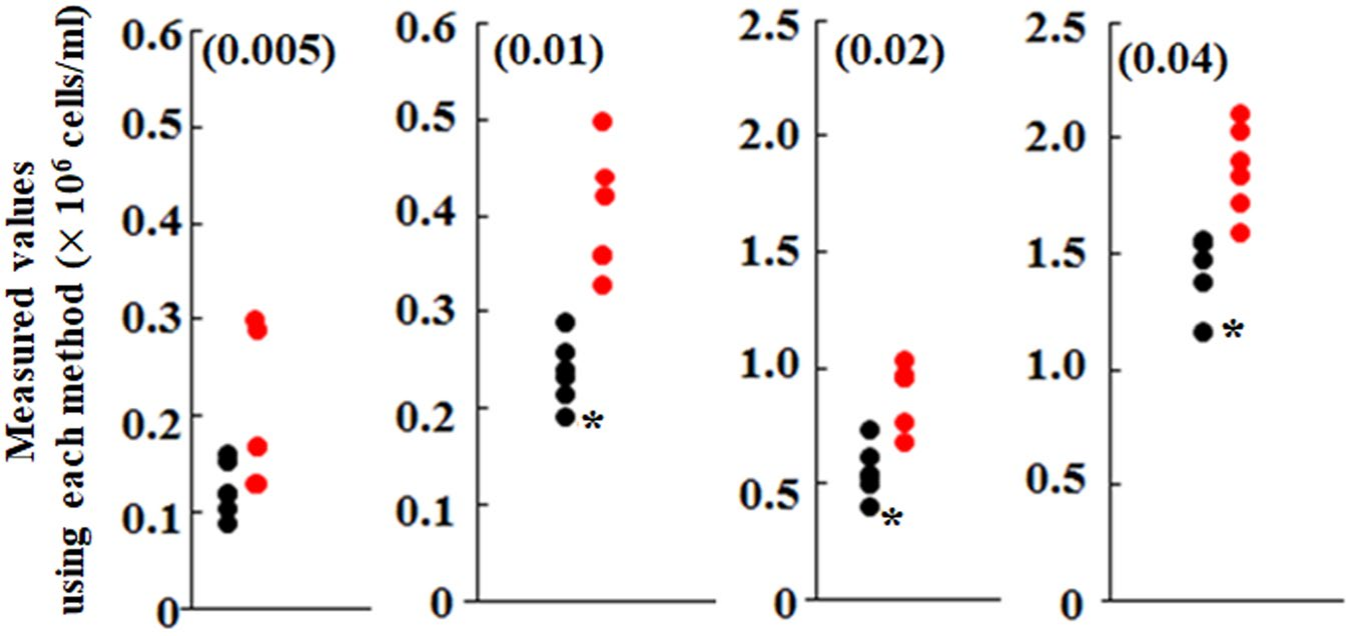
Statistical evaluation on accuracy of measurement values using the cell counter. These panels take *Chlorella*-like alga from *P. bursaria*, for example. To evaluate whether each value using the cell counter (black circle) is included in the variation from hemocytometry (red circle), values from the cell counter were subjected to Smirnov–Grubbs outlier testing. An asterisk denotes an outlier value.

To evaluate whether each measurement value using the cell counter is admissible as an alternative from hemocytometry, a Smirnov–Grubbs outlier test was applied to all values obtained using the cell counter. Figure 5 presents results obtained using *Chlorella*-like algae as an example of the Smirnov–Grubbs outlier test. Although only a few values of outliers (asterisks) to the variation from hemocytometry were detected from data obtained using the cell counter, almost every value from the cell counter fell within the variation of data using hemocytometry. Data other than those of *Chlorella*-like algae showed the same tendency.

**Figure 6:**
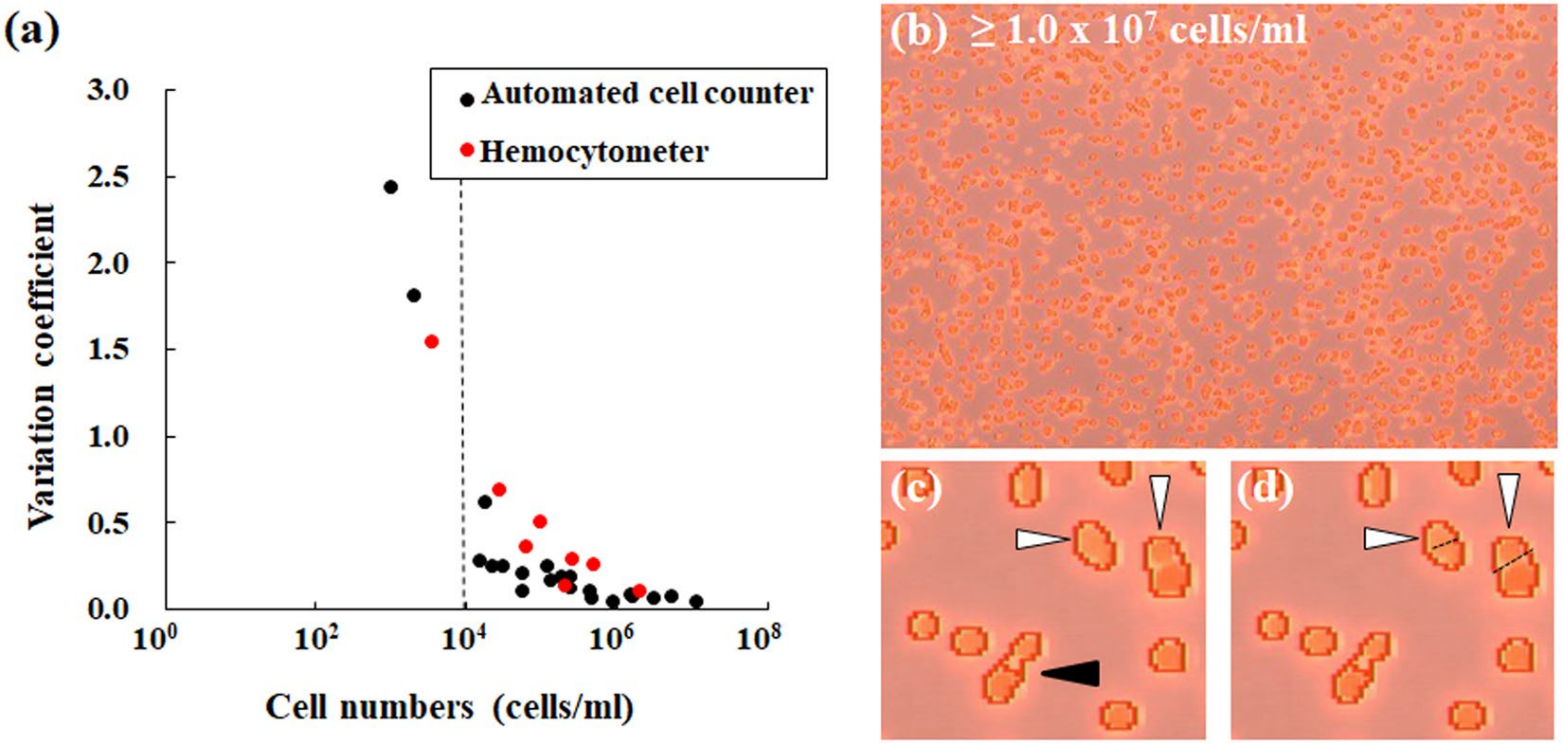
Measurement accuracies of the cell counter and a hemocytometer. (**a**) The CV obtained from the cell counter (black circle) was compared with that from a hemocytometer (red circle) by density. (**b**) Image taken at cell density rather than 10^7^ cells/ml. (**c** and **d**) These photographs present enlarged views of panel (b). By default, images (panels (b–d)) from the cell counter have no scale bar.

For comparison of the respective accuracies of cell density measurements obtained using the cell counter and the hemocytometer, the relations between cell numbers and the corresponding coefficient of variation (CV) are shown for the respective methods (Fig. 6a). Here, the values obtained using the cell counter (black circle) are data obtained using the fluorescence filter. The CV of almost all values obtained using the cell counter tended to be lower than that obtained using the hemocytometer (red circle). Each CV value, however, was drastically higher under conditions of microalgae at low densities less than 10^4^ cells/ml, whichever method was selected. From image analysis using the attached camera in conditions with density of microalgae higher than 10^7^ cells/ml, overlapping of several algae was also observed (Fig. 6b–d). Although each particle indicated by a white arrowhead (Fig. 6c), for instance, was detected as 1 cell by default, these particles must be recognized as two cells (Fig. 6d). Consequently, detailed analysis using images (Fig. 6c–d) indicated the potential for increased underestimation of the algal number in conditions with high algae density.

### Applicability of the cell counter to algal status evaluation based on chlorophyll integrity

**Figure 7:**
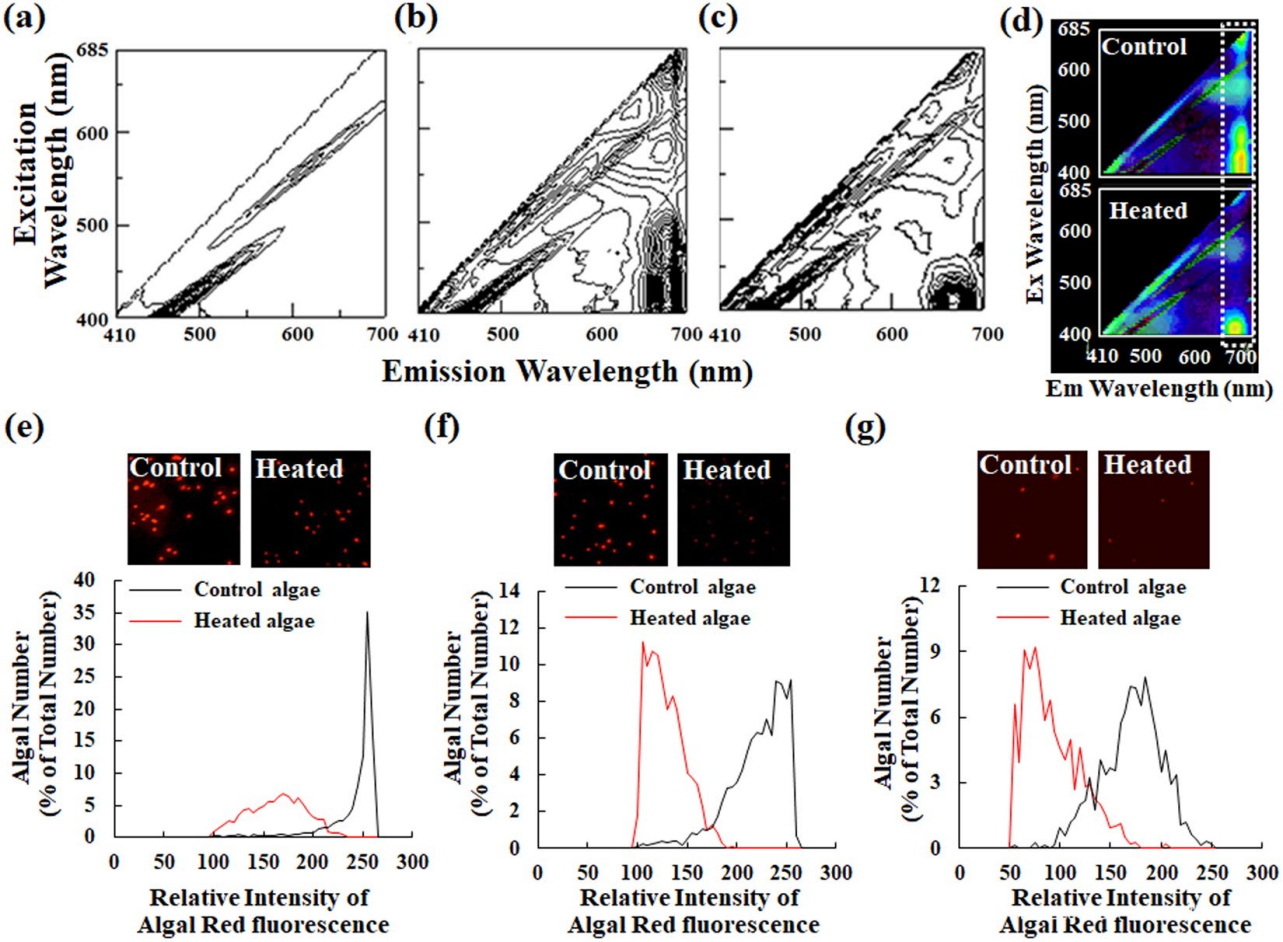
Detection of algal status using the cell counter. (**a**–**c**) These graphs show 3D fluorescence excitation-emission matrix spectrographs of a medium solution and *Chlorella*-like algae with or without heat treatment. (**d**) Difference spectra between the 3D fluorescence excitation-emission matrix image of control algae and that of only medium, and between that of heated algae and that of only medium using software (Adobe Photoshop; Adobe Systems Inc.). Fluorescence signals corresponding approximately to chlorophyll fluorescence are enclosed within a white dotted line. (**e**–**g**) Changes of algal status were evaluated using the cell counter before (black line) and after heat treatment (red line) of each alga. Panels (e),(f), and (g) respectively present results for *P. kessleri*, *Chlorella*-like algae, and sea algae. Signals show more than 100 relative fluorescence for *P. kessleri* and *Chlorella*-like algae and those with more than 50 relative fluorescence for sea algae.

Several microalgae are extremely sensitive to various organic and inorganic pollutants that can affect algal metabolisms^10^. Moreover, chlorophyll is sensitive to changes in temperature and pH (e.g., heat and acidic environment), which deactivate and degrade chlorophyll^16,19,20^. The excitation and emission spectra after heat treatment of *Chlorella*-like algae, as an example, are shown using spectrofluorometry (Fig. 7a–c). Here, heat treatment was used as a positive stress control in this study. The contour plots show that algae without heat treatment (Fig. 7b) had stronger red fluorescence emission derived from chlorophyll near at 680 nm than algae with heat treatment did (Fig. 7c). To test whether algae under the stress condition have significant chlorophyll fluorescence, difference spectra were performed respectively between the 3D fluorescence excitation-emission matrix image of control algae and that of only medium, and between that of heated algae and that of medium (Fig. 7d). In principle, the 3D fluorescence matrix image after the difference spectrum must be blackened if a spectrum is completely consistent with the other one. The difference spectra show that algae have marked chlorophyll fluorescence (white dotted box in Fig. 7d) even if under the stress condition. Consequently, the red fluorescence of algae can serve as an algal status indicator in routine applications. This cell counter can evaluate not only the number of cells but also an arbitrary fluorescence property of cells. This study examined whether the cell counter evaluates changes of algal status based on chlorophyll fluorescence after heat treatment of algae (Fig. 7e–g). Comparison of the fluorescence intensity of control algae without heat treatment and heated algae revealed that all types of heated microalgae exhibited drastically attenuated red fluorescence (inset images in Fig. 7e–g). Histographic analysis of data obtained using the cell counter revealed that heat treatment of all microalgae drastically altered their fluorescence from high intensity to low intensity (Fig. 7e–g). These results demonstrate that the cell counter can evaluate the algal status.

### Detection capability of amorphous phytoplankton other than spherically shaped alga using the automated cell counter

**Figure 8:**
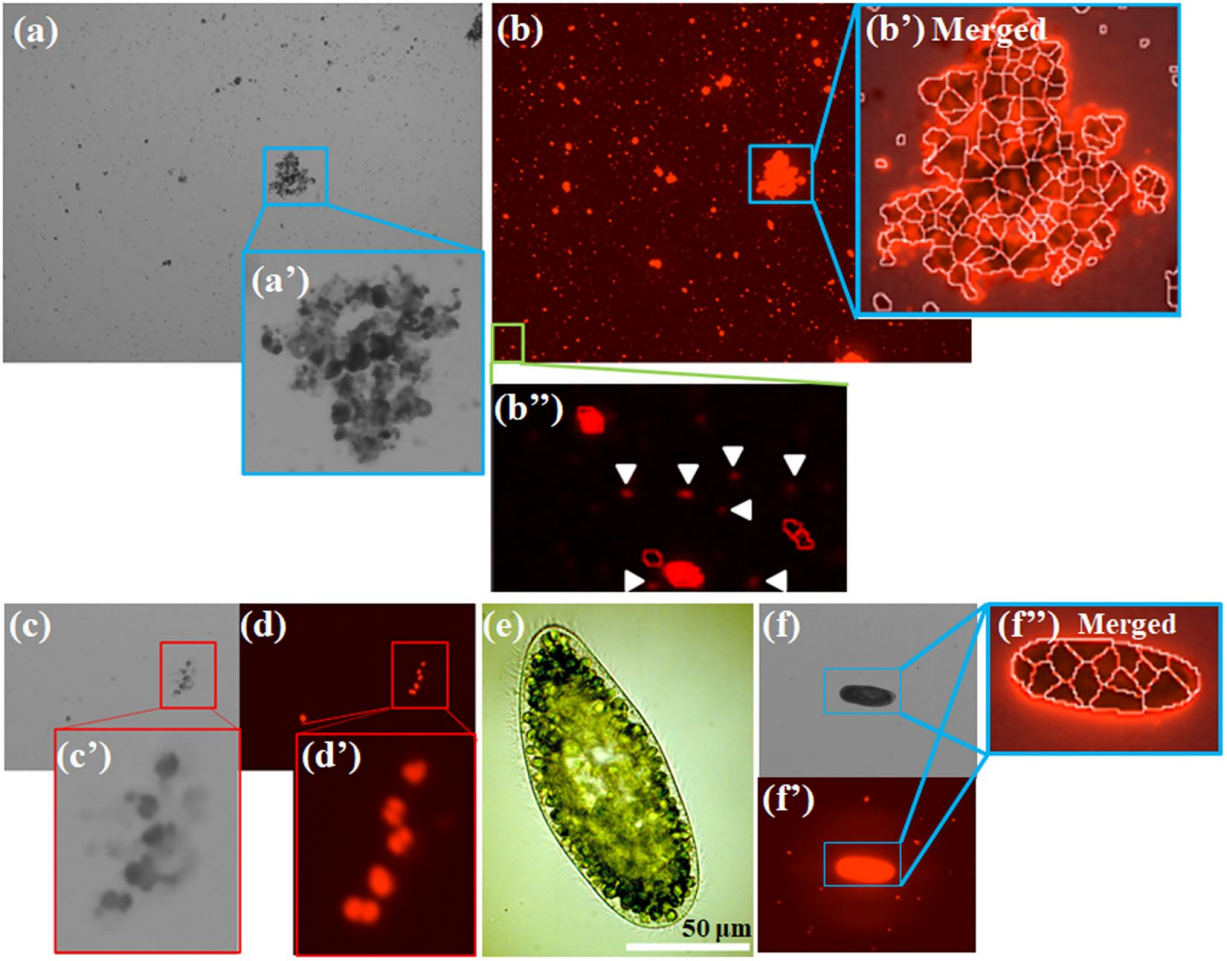
Detection of photosynthetic microbes other than cultured unicellular algae using the cell counter. A pond sample as an environmental sample is shown in panel (**a**) and the corresponding fluorescence image (**b**). Panels (a’), (b’) and (b”) respectively portray magnified images of panel (a) and (b). Panel (b’) depicts a merged image of the bright image with the corresponding fluorescence image. A river sample is also shown in panel (**c**) and the corresponding fluorescence image (**d**). Panels (c’) and (d’) respectively depict magnified images of panels (c) and (d). (**e**) Micrograph of *P. bursaria* using light microscopy, but not the cell counter. A bright field image (**f**), the corresponding fluorescence image (f’), and both merged image (f”) of *P. bursaria* from the cell counter are shown. Although a single *P. bursaria* cell is shown in both the bright field and the corresponding fluorescence image, the cell was recognized falsely as clumps of cells. By default, no original image from the cell counter has a scale bar. To make algae more visible, each image is shown at arbitrary magnification.

Some spherical unicellular algae including *Dunaliella salina*, *Chlorella* sp., *Haematococcus pluvialis* and *Coelastrella striolata var. multistriata* have been used as valuable algae for industrial use to produce canthaxanthin, astaxanthin, α-carotene and β-carotene, lutein, neoxanthin, violaxanthin, zeaxanthin, echinenone and so on^21,22^. For instance, other valuable algae such as *Botryococcus braunii*^23^ to produce biofuels and *Arthrospira* sp.^22^ to produce astaxanthin, zeaxanthin, oscillaxanthin, lutein, β-carotene, echinenone and myxoxanthophyll are amorphous or filamentous algae. It particularly remains unclear whether the cell counter is able to precisely detect amorphous phytoplankton other than those having a globular shape. To check the capability of the instrument for microalgae detection, environmental samples including microalgae and non-spherical plankton *P. bursaria* with symbiotic algae were examined using the instrument. Although it is too difficult to carry out species identification using the low-resolution camera alone, microalgae such as *Microcystis* sp. forming cell colonies were detected morphologically in the pond sample (Fig. 8a,a’,b and b’). An individually distinguished alga is shown in the merged image (Fig. 8b’) of the bright field image (Fig. 8a) into the corresponding fluorescence image (Fig. 8b) using the specified algorithm of the instrument. Although Fig. 8b” presents unfocused cells (white arrows) with clear red fluorescence, the cell counter did not detect and count these red signals because the cells were too small. Moreover, microalgae such as *Tetraspora* sp. were detected morphologically in the Okimizu River sample (Fig. 8c,8c’, 8D and 8d’), although it was an imprecise species identification. It is striking that an alga close to other alga was also distinguished definitely.

In contrast to the results of environmental samples presented above, measurement of *P. bursaira* using the cell counter reached an anticlimax with the unsatisfactory result (Fig. 8). Although *P. bursaira* has several hundred endosymbiotic algae in the cytoplasm (Fig. 8e), *P. bursaira* cells appeared as a large lump in images taken by the camera of the cell counter: both bright field and fluorescence images (Fig. 8f and f’). The cell counter, however, judged the paramecium cell as clumpy cells (Fig. 8f”).

## Discussion

This study examined the applicability of a cell counter (Countess II FL; Thermo Fisher Scientific Inc.) to detection of microalgae and to evaluation of their algal status. This study used the fluorescence filter only for Cy5, which can detect algal fluorescence derived from chlorophyll (Fig. 2b–c). Therefore, the cell counter used for this study had only one chlorophyll-detection function. Additional and simultaneous use of other fluorescence filters also enables detection of cellular and metabolic parameters other than chlorophyll using fluorescently labeled markers. Reportedly, the cell walls of algae differ in their permeability to dyes^24^. For instance, to visualize DNA in some algal species using DAPI, a particular chemical (e.g., toluene, SDS) or physical (e.g., freezing, microwave) pretreatment of algae must be used to improve the permeability of organic molecule dyes to algal cell walls^24^. As an example, Supplemental Fig. 2 shows the difference between *Chlorella*-like symbiotic algae and the host *P. bursaria* in terms of the DAPI permeability. In spite of the fluorescence of algal chlorophyll and the host paramecium DNA, no algal DNA was observed because of the low permeability of their algae to DAPI. Although it is necessary to maintain healthy microalgae and to check the algal integrity in industrial applications, a great deal of exertion must be put forth to ascertain an adequate permeable pretreatment for each alga species. In contrast to methods that incorporate the use of organic dye, detection of chlorophyll is easy because almost all algae have their organelles spontaneously. Therefore, this study specifically evaluated the number of algae and evaluated their algal status based on chlorophyll fluorescence rather than on other parameters such as DNA contents and lipids.

To avoid false recognition of objects other than microalgae by the cell counter, each threshold level of target algae was ascertained carefully by observation of the practical real-time detection image of each alga using the instrument. Commercially available automated cell counters have a monochromatic camera, which typically has lower resolution than that used for common microscopic observation. It is challenging for these cameras to discriminate small cells such as microalgae from other particles such as debris, small stains, and spots in a counting chamber. If microbubbles, which typically have 1–10 μm diameter^25^, occur at the time of enclosing cells within the counting chamber, then they might cause false recognition as small cells. In fact, the cell counter used for this study also detected objects other than non-fluorescent ones using size selection alone (Fig. 2e and e’). To detect microalgae accurately using the cell counter, fluorescent selection is useful to discriminate microalgae from other particles (Fig. 2f). Results of this study demonstrate that the cell counter detected microalgae precisely by discriminating the chlorophyll fluorescence of microalgae (Figs 2c and 3a–d) if the algal size is less than that of the cultured animal cells (Fig. 3e).

In addition to evaluation of the number of cells, the cell counter used for this study can elucidate the cell size and arbitrary fluorescence properties of interest simultaneously. Once the cell counter detects microalgae without false recognition (Fig. 3d), size evaluation of microalgae is also regarded as reasonable and proper as that using microscopic measurement (Fig. 3e–f). This cell counter was designed originally as an alternative to a hemocytometer for counting cells of mammalian cell lines and hemocytes rather than microalgae. When passage cultures of floating cells such as hemocytes and adhesive cells treated with trypsin are conducted, hemocytometry has been conventionally used. These cells are globular even if in the case of adhesive cells trypsinized and scratched for passage culture. Therefore, the size evaluation of cells using the cell counter is regarded as evaluation of the cell diameter. In fact, values of the algal size using the cell counter are equivalent to the diameter of algae using manual image analysis (Fig. 3g).

Although values obtained using the cell counter with the fluorescence filter were evaluated statistically as similar to those obtained using hemocytometry (Figs 4 and 5), values obtained using the cell counter without the fluorescence filter occasionally included false recognition of particles other than algae (Fig. 4). As described above, this result indicates that fluorescence detection and selection are at least necessary to recognize microalgae precisely. The cell counter measurement principle is identical to that of a hemocytometer. However, it stands to reason that CV values obtained using the cell counter tended to be lower than those obtained using the hemocytometer (Fig. 6) because measurements using the cell counter have a wider measurement area (3.48 mm^2^) than that using a conventional hemocytometer (1 mm^2^). Analogously with the method using conventional hemocytometry, each CV value increased significantly when the algal density was lower than ca. 10^4^ cells/ml (Fig. 6). Excess algal density (>ca. 10^7^ cells/ml) was also inadequate for determination of an algal number because of the overlap of cells (Fig. 6b–d). This overlapping of algae might cause false recognition of each alga. Considering CV values and the overlapping of cells, cell density of ca. 10^5^–10^6^ cells/ml might be a desirable condition for the cell counter to detect cells precisely, analogously with conditions of general hemocytometry.

Algal metabolism is affected also by trace levels of contaminants, including various organic and inorganic pollutants such as heavy metals^10^. Such factors might pose a threat to algal culture. Chlorophyll is also sensitive to heat treatment (Fig. 7a–c) because thermal stress damages the thylakoid membrane, which is related to structural and functional changes of the photosystem (PS) II and PS I, and to interruption of the Calvin cycle^26,27^. Using red fluorescence derived from chlorophyll as an indicator of algal status (Fig. 7d), this study examined whether the cell counter evaluates changes of algal status based on chlorophyll integrity (Fig. 7e–g). All algae treated with heat showed drastically reduced red fluorescence (Fig. 7e–g). Consequently, the cell counter was able to evaluate the algal status. Takahashi *et al*. used yellow fluorescence derived from degradation of chlorophyll to evaluate dying or dead algae^16^. To evaluate metabolic status in algae other than that of chlorophyll, the other parameters and the corresponding fluorescence filter might also be useful if assisted by arbitrary fluorescent dyes to monitor metabolism. Algal size might sometimes change depending on stress conditions such as nitrogen starvation and high light stress. Describing the following comments just in case, the cell counter has excellent flexibility for obtaining data. Although this study examined the algal status according to each preset threshold level, out-of-range values are also measured automatically and recorded. In addition, one can change the threshold level as necessary during cell counting estimation.

Valuable microalgae are not always spherical and unicellular. Some valuable algae are amorphous or filamentous. As described above, this cell counter was designed mainly for mammalian cell lines. When microalgae forming a cell colony or clumps were analyzed using the cell counter, an individual cell in a highly complicated clump (Fig. 8a,b and b’) was distinguished according to the instrument algorithms. Even if using manual cell counting as with hemocytometry, accurate cell counts of samples containing clumpy cells are difficult to obtain. Therefore, the accuracy to identify an individual alga in clumpy cells was unclear. Individual cells in simple cell clusters of fewer than 10 cells (Figs. 8c and 8d) appeared to be captured adequately using the cell counter. Extremely small cells were recognized inappropriately using the cell counter (Fig. 8b”). A bacterial counter similar to a hemocytometer has been used for the manual counting of bacteria using microscopy. The depth of a standard bacterial counter is 20 μm, whereas that of a standard hemocytometer such as a Thoma hemocytometer is 100 μm. The chamber space of the cell counter designed for mammalian cells must be sufficiently large for these small cells less than 1 μm such as small cyanobacteria. Overly large cells also seem to be unsuitable for counting by the cell counter. *P. bursaria* (Fig. 8e), for instance, has cell width and length of *ca*. 50 μm and 120–150 μm. A range of the cell counter in size is up to 60 μm, which can cover sizes of almost all mammalian cell lines but which is entirely inadequate for some largish plankton. The result is that *P. bursaria* cell was regarded as clumpy cells using the cell counter (Fig. 8f”). Considering these features of the cell counter on detection of phytoplankton using chlorophyll fluorescence, it might be difficult for the cell counter to precisely recognize coenobium plankton containing a fixed number of cells like *Volvox* sp. and *Scenedesmus* sp. as their cluster even if the plankton size is an acceptable range of the cell counter. The cell counter might detect 1 μm of an object. In contrast to a largish cell such as *P. bursaria*, approximately 1 μm of cyanobacteria and fluorescence-labeled bacteria might be estimable cells if an adequate chamber with low depth such as a bacterial counter is prepared.

In conclusion, the automated cell counter with a fluorescence filter for chlorophyll can precisely distinguish chlorophyll-bearing unicellular cells from other particles. It is useful to ascertain the number of algae, the algal size, and their status based on chlorophyll integrity. This instrument can save greater amounts of time than conventional microscope-based evaluation methods such as hemocytometry. The potential of this cell counter without user bias is useful for routine management of algal cultures in laboratory and industrial applications by detecting aberrant algae. Taken together, results show that this system can contribute to algal application as a powerful tool. In addition to these featured functions, the compact form of the cell counter can make it easy to take anywhere. The portability of this instrument might also contribute to *in situ* environmental surveys as a simplified test to measure algae related to algal blooms.

## Materials and Methods

### Algae preparation

*Parachlorella kessleri* (C-531 strain) as *Chlorella kessleri* was obtained from the Institute of Applied Microbiology (IAM) culture collection at The University of Tokyo. Based on a combination of classical and modern methods including molecular phylogeny and bioinformatics, the scientific name of *C. kessleri* was changed recently to *P. kessleri*^28^. The algae were cultured on agar plates containing CA medium^29^. *Chlorella*-like symbiotic algae (SA-1) isolated from *P. bursaria*^29^ were used. The cloned strain of symbiotic algae on an agar plate containing CA medium was cultured separately from the host *Paramecium*. Sea algae species for live bait of sea fish were purchased from Nikkai Center Ltd. (Tokyo, Japan). The sea algae were cultured in liquid f/2 medium. Microalgae of three kinds were mainly used for this study as model freshwater and marine microalgae. These algae were cultured under an LD cycle (12 hr light/ 12 hr dark) with 1100 lux of natural white fluorescent light and 23 ± 2 °C. Before experiments, these algae of *P. kessleri* and *Chlorella*-like symbiotic algae on the plates were scratched with an inoculating needle. Subsequently, they were suspended in new liquid CA medium or in ultrapure water. Parts of the sea algae culture were used for later experiments including phylogenetic clustering for sea alga species identification because the sea algae did not originate from any type culture collection of a research institution. Detailed descriptions of DNA extraction, PCR amplification of 18 S rDNA, and phylogenetic clustering for sea alga species identification are presented in *Supplemental Materials*.

### Permeability estimation of *Chlorella*-like symbiotic algae to a DNA binding organic dye (DAPI) using fluorescence microscopy

*P. bursaria* syngen I (BWK-4, mating type IV) collected from Lake Biwa (Shiga, Japan) and *P. bursaria* syngen I (AS-2, mating type IV) collected from the Ashida-kawa River (Hiroshima prefecture, Japan) were used for this study. Paramecia in a lettuce infusion containing *Klebsiella pneumonia* as food were cultured under an LD cycle (12 hr light/12 hr dark) at 1500 lux of natural white fluorescent light and 23 °C*. P. bursaria* was harvested using a pipette and placed on a coverslip. The paramecia were fixed with methanol for 6 hr at −20 °C^30^. The fixed cells were washed twice using PBS and were mounted in FluoroGuard (Bio-Rad Laboratories Inc.) containing DAPI (Nacalai Tesque Inc.) for DNA staining. Specimens containing paramecia were observed using Nomarski difference contrast and fluorescence microscopy (Optiphot; Nikon Corp., Tokyo, Japan).

### Confirmation of each fluorescence filter’s propriety for a cell counter

A commercially available Countess II FL cell counter (228.6 mm [width] × 139.7 mm [depth] × 228.6 mm [height]) (Thermo Fisher Scientific Inc.) equipped with an arbitrary fluorescence filter was used to detect microalgae. The cell counter has a 5-megapixel digital and monochromatic camera with 2.5 × optical zoom. Before determination of the number of algae using the cell counter, the propriety of the fluorescence filter for the cell counter was examined first using online software (SpectraViewer; Thermo Fisher Scientific Inc.). To confirm the propriety of the fluorescence filter experimentally, both excitation and emission spectra of *Chlorella*-like symbiotic algae adjusted to 1.0 × 10^7^ cells/ml were analyzed using a spectrofluorometer (FP-8200; Jasco Corp.).

### Cell size evaluation of algae using the automated and imaging-based cell counter

This study used a cell counter equipped with a fluorescence filter (Ex 628/40, Em 692/40; EVOS Light Cube for Cy5; Thermo Fisher Scientific Inc.) and used a dedicated reusable slide. To avoid false recognition of non-alga particle as algae by the cell counter, one can set an arbitrary size threshold level (size of 0–60 μm) and the corresponding fluorescence intensity (relative intensity of 0–255) with respect to each assay. Each threshold level of target algae was ascertained by reference to the cell size of several previous reports of the literature for each target alga, and by observation of the practical real-time detection image of each alga using the instrument. In addition to detection of algae using this cell counter, the algal size was measured simultaneously. All data were expressed as an average ± standard deviation. To confirm the validity of algal size using the cell counter, algae were also observed using light microscopy. Here, values of algal size using the cell counter were compared with those using both microscopy and manual image analysis. Manual determination of algal size was performed using software (ImageJ). The area and circularity of individual alga and the length of the pixel size per micrometer were ascertained respectively using the software. The diameter of each alga was calculated using the following equation (1).

1 $${\rm{Algaldiameter}}=2\times \sqrt{\frac{Algal\,area}{\pi }}\times \frac{Micrometer\,length\,(\mu m)}{pixcel\,size}$$

### Accuracy verification of the automated cell counter on cell count using Smirnov–Grubbs outlier testing

Arbitrary concentrations of diluted algal samples were prepared. Then, the cell density of each sample was ascertained using both the cell counter and a hemocytometer. The counted values obtained using the cell counter were compared with those obtained using the hemocytometer. All data were expressed as the average ± standard deviation.

To assess the accuracy of a counting value obtained using the cell counter and to evaluate whether each value is included in the variation from hemocytometry, the values from the cell counter were subjected to Smirnov–Grubbs outlier testing. Each value derived from Smirnov–Grubbs test was calculated using the following equations (2)–(4).

2 $$T=|\frac{{C}_{c}-avg.}{SD}|$$

3 $${t}_{0}=tinv(N-2,\,\frac{2\alpha }{N})$$

4 $${S}_{{0}}=(N-1)\sqrt{\frac{{t}_{0}^{2}}{N(N-2)+N{t}_{0}^{2}}}.$$

In those equations, *T* and *Cc* respectively represent the test statistic and arbitrary value obtained using the cell counter in equation (2) above. In addition, *avg*. and *SD* respectively denote the average and standard deviation, calculated respectively from values of *Cc* and values obtained using hemocytometry. Also, *t*_0_ denotes the value of the *t*-distribution with *N*−2 degrees of freedom and 2α/*N* in equation (3) above. *N* denotes the number of data composed of an arbitrary value obtained using the cell counter and data obtained using hemocytometry (6 in this study). For this study, α = 0.05 was used to infer significance. In equation (4) above, *S*_0_ was used as a reference point. When a *T* value in equation (2) is greater than the corresponding *S*_0_ value in equation (4), the value used from the cell counter was ascribed to an outlier. To assess the accuracy of a counting value using the cell counter and the hemocytometer, the coefficient of variation (CV) was also determined.

### Cell status evaluation of algae using the automated cell counter

To confirm the effectiveness of the cell counter for the assessment of algal status as chlorophyll integrity, dead or dying algae were prepared by heat treatment of algae for 10 min at 100 °C. To assess heat stress effects on algae, both excitation and emission spectra of algae treated with or without heat were analyzed using spectrofluorometry. It was first ascertained whether significant fluorescence from chlorophyll is detected even if after treatment of algae with heat. Briefly, difference spectra between the 3D fluorescence excitation-emission matrix image of control algae and that of only medium, and between that of heated algae and that of medium were performed as image analysis using software (Adobe Photoshop; Adobe Systems Inc.).

After heat treatment of algae, the algal status based on Cy5-like red fluorescence was measured using this cell counter. Histograms of algae before and after heat treatment were compared. We digitized data points (graph digitizing system, GSYS2.4 software; Hokkaido University Nuclear Reaction Data Center) for each figure in the form of a graphical image from the cell counter. Moreover, to remove noise signals derived from non-cell debris and microbubbles, red fluorescence signals with greater than relative fluorescence intensity of 100 in *P. kessleri* and *Chlorella*-like symbiotic algae were estimated. Those with relative fluorescence intensity greater than 50 in sea algae were also counted using this cell counter.

### Scalability verification of the automated cell counter on detectable alga species and photosynthetic plankton

To test the automated cell counter for applicability of detectable algae or plankton with photosynthetic pigments, environmental samples or a culture sample of *P. bursaria* were used. Approximately 500 ml of a pond sample was collected from a pond at the National Institute of Technology, Miyakonojo College, Miyazaki, Japan. Moreover, an approximately 4300 ml environmental sample was collected from the Okimizu River at Miyakonojo, Miyazaki, Japan (31°45′01.3′′N, 131°04′38.0′′E). Those environmental samples were centrifuged and condensed into 10 ml. The red fluorescence of chlorophyll-bearing cells in the concentrated samples was detected using the cell counter. Then 100 ml of the culture of *P. bursaria* (AS-2 strain) was collected and used for detection using the cell counter. For measurements of *P. bursaria*, *P. bursaria* was fixed with 10% (v/v) formalin for inhibition of its high mobility. Samples containing *P. bursaria* were analyzed using the cell counter and a dedicated disposable slide.

## Electronic supplementary material

Supplemental Figures 1 and 2
