# Supplementary material for: Applicability of Automated Cell Counter with a Chlorophyll Detector in Routine Management of Microalgae
Source: Sci Rep. 2018 Mar 21;8:4967. doi: 10.1038/s41598-018-23311-8 (PMC5862891; doi:10.1038/s41598-018-23311-8)
Supplement: Supplementary file 1 — Supplemental Figures 1 and 2 [file 41598_2018_23311_MOESM1_ESM.pdf]

Applicability of Automated Cell Counter with a Chlorophyll Detector in Routine Management of Microalgae

Toshiyuki Takahashi*

Department of Chemical Science and Engineering, National Institute of Technology, Miyakonojo College, Japan

*Correspondence author: mttaka@cc.miyakonojo-nct.ac.jp

Telephone number: +81-986-47-1219; Fax number: +81-986-47-1231

Grant sponsors: Grant-in-Aid for Exploratory Research from Japan Society for the Promotion of Science (KAKENHI Grant Numbers 23658280 and 17K05955)

Supplemental material

DNA extraction, PCR amplification of 18S rDNA, and phylogenetic clustering for sea algae species identification. This study confirmed species identification for the sea algae by 18S rDNA amplification because the sea algae did not originate from any type culture collection of a research institution. DNA from the sea algae were extracted using the DNAeasy Plant kit (Qiagen Inc.). First, the sea algal sample was centrifuged and collected in a 2-ml tube containing glass beads (GMB-60; Nippon Rikagaku Kikai Co., Ltd.). The sea algal pellet was then homogenized twice for 60 s at 4000 rpm in a mini-bead cell disruptor (μT-01; Taitec Corp.). The homogenate sample was processed according to the manufacturer's instructions for the DNAeasy Plant kit. The following primer pair for 18S rRNA gene sequences of eukaryotic microalgae was prepared: 5′-cgactagccaatggaagcat-3′ for the forward primer and 5′-gtacaaagggcagggacgta-3′ for the reverse primer. The primer pair was used for PCR. PCR amplification from 10 ng of sea algal DNA was conducted using the Quick Taq HS DyeMix (Takara Bio Inc.) under the following amplification conditions: 2 min at 94 °C, 40 cycles of 30 s at 94 °C, 30 s at 50 °C, and 1-min extension step at 68 °C. To ascertain whether the PCR product is a single band, or not, the PCR product was separated using electrophoresis on a 2.0% agarose gel stained with SYBR Green. Fragments such as primers in the PCR product were eliminated using a HiYield™ Gel/PCR DNA Fragment Extraction Kit (RBC Bioscience Corp.) for the DNA sequence. The DNA sequencing was outsourced to Fasmac Co. Ltd. (Japan). The phylogenetic relation of the partial 18S rDNA (GenBank: LC314323.1) was ascertained through a distance tree created using maximum likelihood method (MEGA 7 software). Here, sequences against which the sea algae were compared were the following: 18S rRNA genes of uncultured stramenopile clone 6c-C6 (GenBank: FN690661.1), *Spumella elongata* (GenBank: AJ236859.1), *Mallomonas papillosa* (GenBank: HF549062.1), *Paraphysomonas sp.* 22 JMS-2012 (GenBank: JQ967330.1), *Ochromonas sp.* CCMP1278 (GenBank: U42382.1), *Heterothrix debilis* (GenBank: U43277.1), *Botrydium stoloniferum* (GenBank: U41648.1), *Tribonema aequale* voucher UTEX (GenBank: HQ710588.1), *Botrydiopsis intercedens* (GenBank: U41647.1), *Heterococcus caespitosus* strain SAG

835-2a (GenBank: AM490820.1), *Dictyota dichotoma* 1CH (GenBank: AF350227.1), *Saccorhiza polyschides* (GenBank: L43059.1), *Pseudostaurastrum limneticum* strain SAG 14.94 (GenBank: EF044313.1), *P. enorme* strain SAG 11.85 (GenBank: EF044312.1), *Vischeria helvetica* UTEX49 (GenBank: AF045051.1), *Eustigmatos magna* (GenBank: U41051.1), *Pseudotetraedriella kamillae* strain SAG 2056 (GenBank: EF044311.1), *Monodopsis subterranea* (GenBank: U41054.1), *Pseudocharaciopsis minuta* (GenBank: U41052.1), *Nannochloropsis gaditana* strain: MBIC10418 (GenBank: AB052271.1), *N. salina* strain: MBIC10063 (GenBank: AB183586.1), *N. limnetica* strain AG 18.99 culture-collection SAG:18.9 (GenBank: AF251496.1), *N. oculata* (GenBank: U38902.1) and *N. granulata* strain: MBIC10054 (GenBank: AB183582.1) in the phylum *Stramenopiles*.

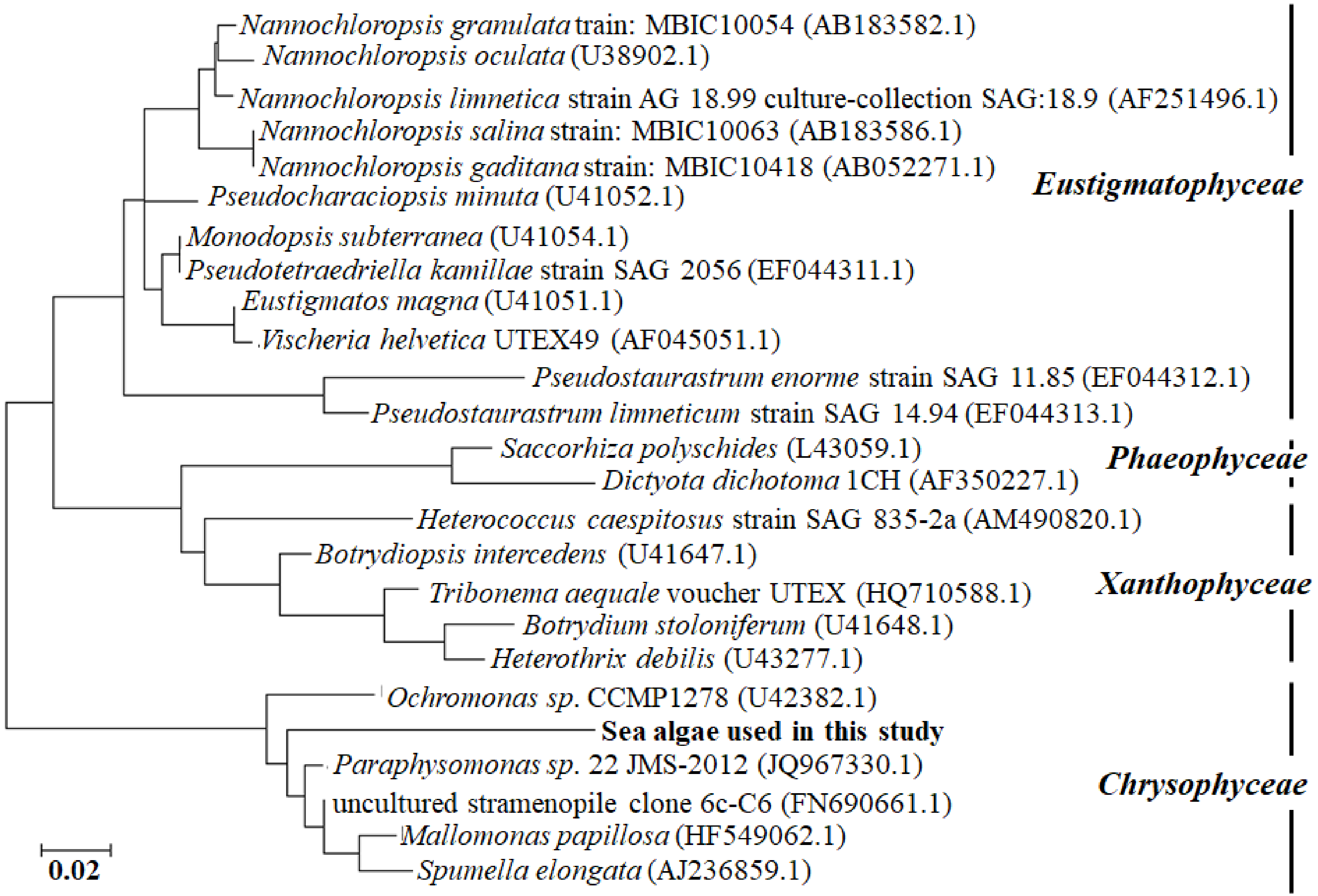

Supplemental Figure 1. PCR amplification and phylogenic analysis of sea algae used for this study. About 200 bps of PCR amplicon (data not shown) from sea algal DNA were amplified. The phylogenic tree was produced from comparison of sea algal 18S rDNA used for this study with other algae in the phylum *Stramenopiles*.

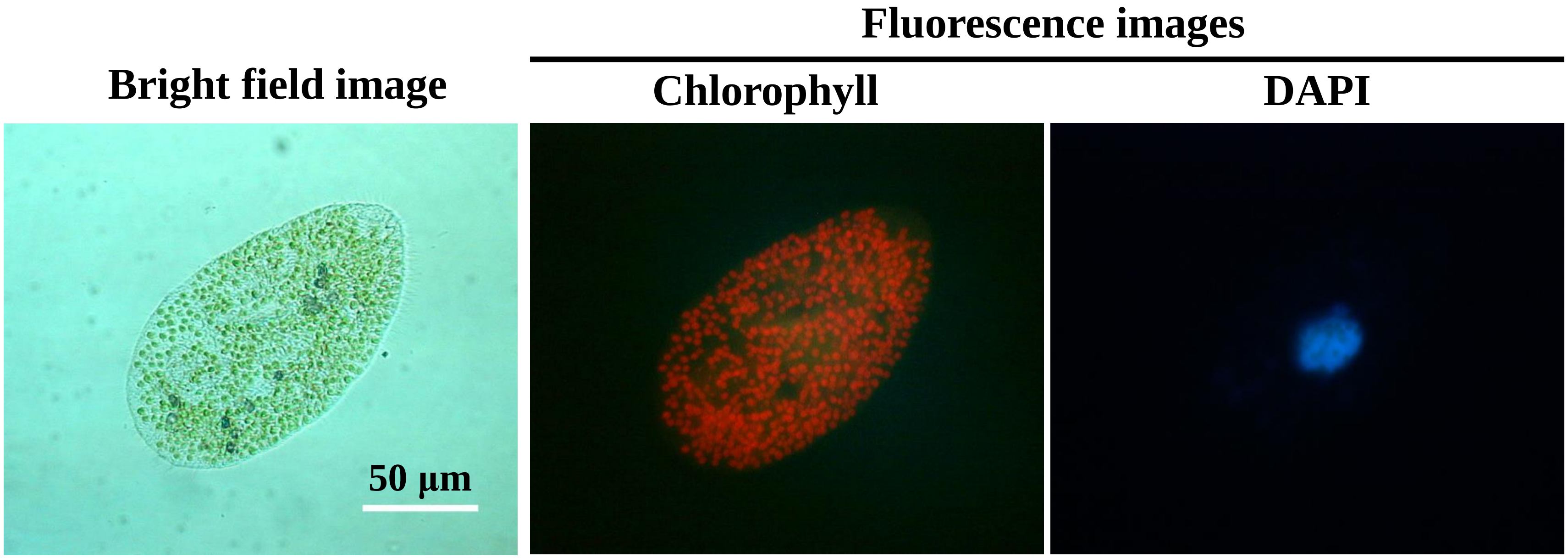

Supplemental Figure 2. Permeability differences between a host *P. bursaria* and the *Chlorella*-like symbiotic algae to DAPI.
